# RETRACTED: Fang et al. MicroRNA-223-3p Regulates Ovarian Cancer Cell Proliferation and Invasion by Targeting SOX11 Expression. *Int. J. Mol. Sci.* 2017, *18*, 1208

**DOI:** 10.3390/ijms26083823

**Published:** 2025-04-18

**Authors:** Gang Fang, Jiao Liu, Qianna Wang, Xueqiong Huang, Runwen Yang, Yuzhou Pang, Meichun Yang

**Affiliations:** Laboratory of Zhuang Medicine Prescriptions Basis and Application Research, Guangxi University of Chinese Medicine, Nanning 530200, China; chizhang0506bira@163.com (G.F.); jicai114277jia@163.com (J.L.); ranggan950128p@163.com (Q.W.); laohuai3329didu@163.com (X.H.); diyue45807chen@163.com (R.Y.); youjiang103670@163.com (Y.P.)

The journal retracts the article titled “MicroRNA-223-3p Regulates Ovarian Cancer Cell Proliferation and Invasion by Targeting SOX11 Expression” [1], cited above.

Following publication, concerns were brought to the attention of the Editorial Office regarding inappropriate image modification and duplication among this publication [1] and earlier articles [2,3] published by different author group.

Adhering to our complaints procedure, the Editorial Office and Editorial Board conducted an investigation that confirmed concerning image modifications and duplication between Figure 3C [1], Figure 4A [2] and Figure 2F [3]. Following discussions with the authors, the Editorial Office, Editorial Board, and authors have decided to retract this article [1] as per MDPI’s retraction policy (https://www.mdpi.com/ethics#_bookmark30).

This retraction was approved by the Editor-in-Chief of the *International Journal of Molecular Science*. 

The authors agreed to this retraction. 
